# Supplementary material for: From Foxtail Millet Husk (Waste) to Bioactive Phenolic Extracts Using Deep Eutectic Solvent Extraction and Evaluation of Antioxidant, Acetylcholinesterase, and α-Glucosidase Inhibitory Activities
Source: Foods. 2023 Mar 8;12(6):1144. doi: 10.3390/foods12061144 (PMC10048580; doi:10.3390/foods12061144)
Supplement: Supplementary file 1 [file foods-12-01144-s001.zip › Figure S2.pdf]

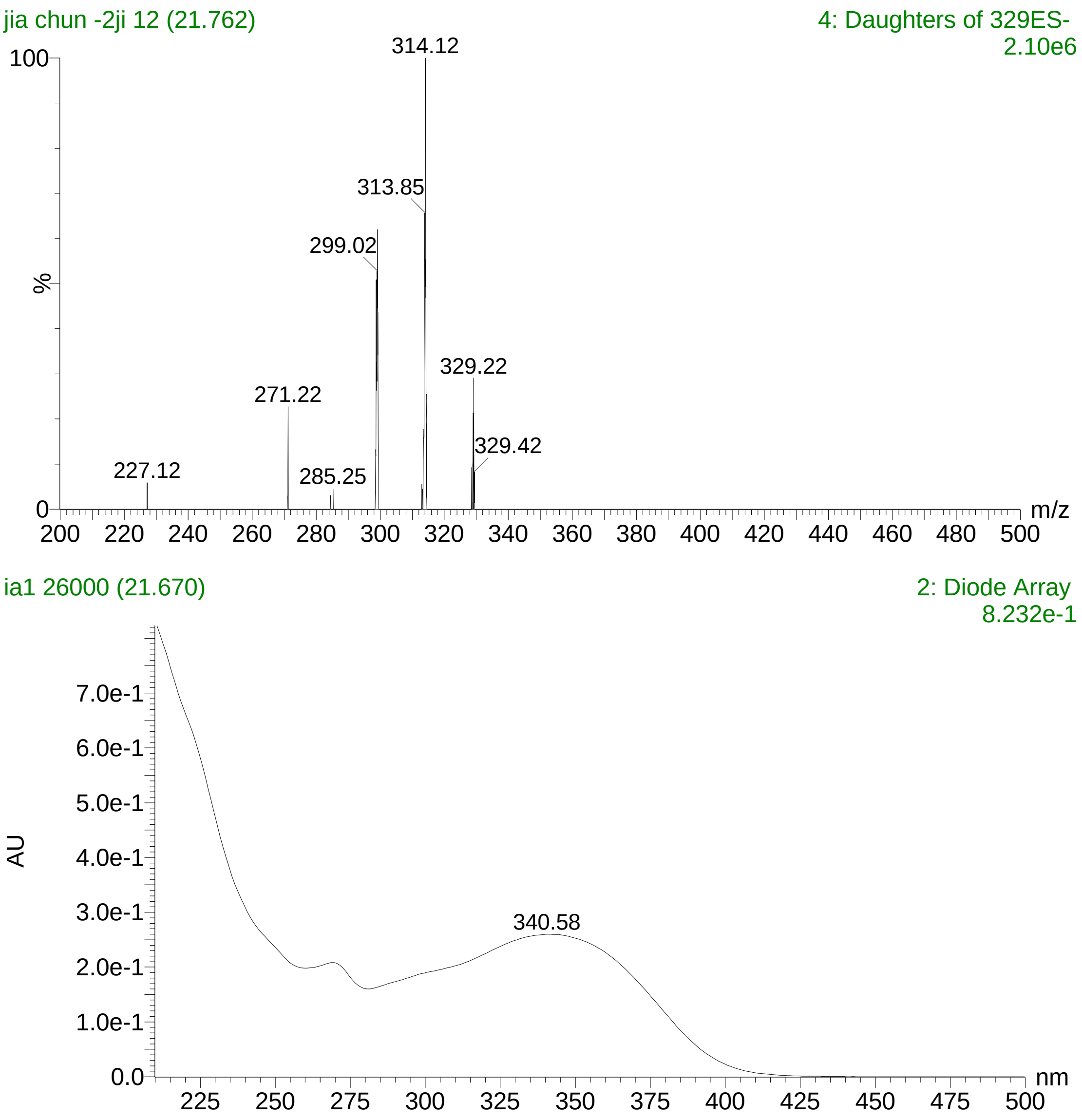

Figure S2. The MS/MS fragments and UV spectrum of 3,7-dimethylquercetin.
