# Supplementary figures and images for: From Foxtail Millet Husk (Waste) to Bioactive Phenolic Extracts Using Deep Eutectic Solvent Extraction and Evaluation of Antioxidant, Acetylcholinesterase, and α-Glucosidase Inhibitory Activities
Source: Foods. 2023 Mar 8;12(6):1144. doi: 10.3390/foods12061144 (PMC10048580; doi:10.3390/foods12061144)

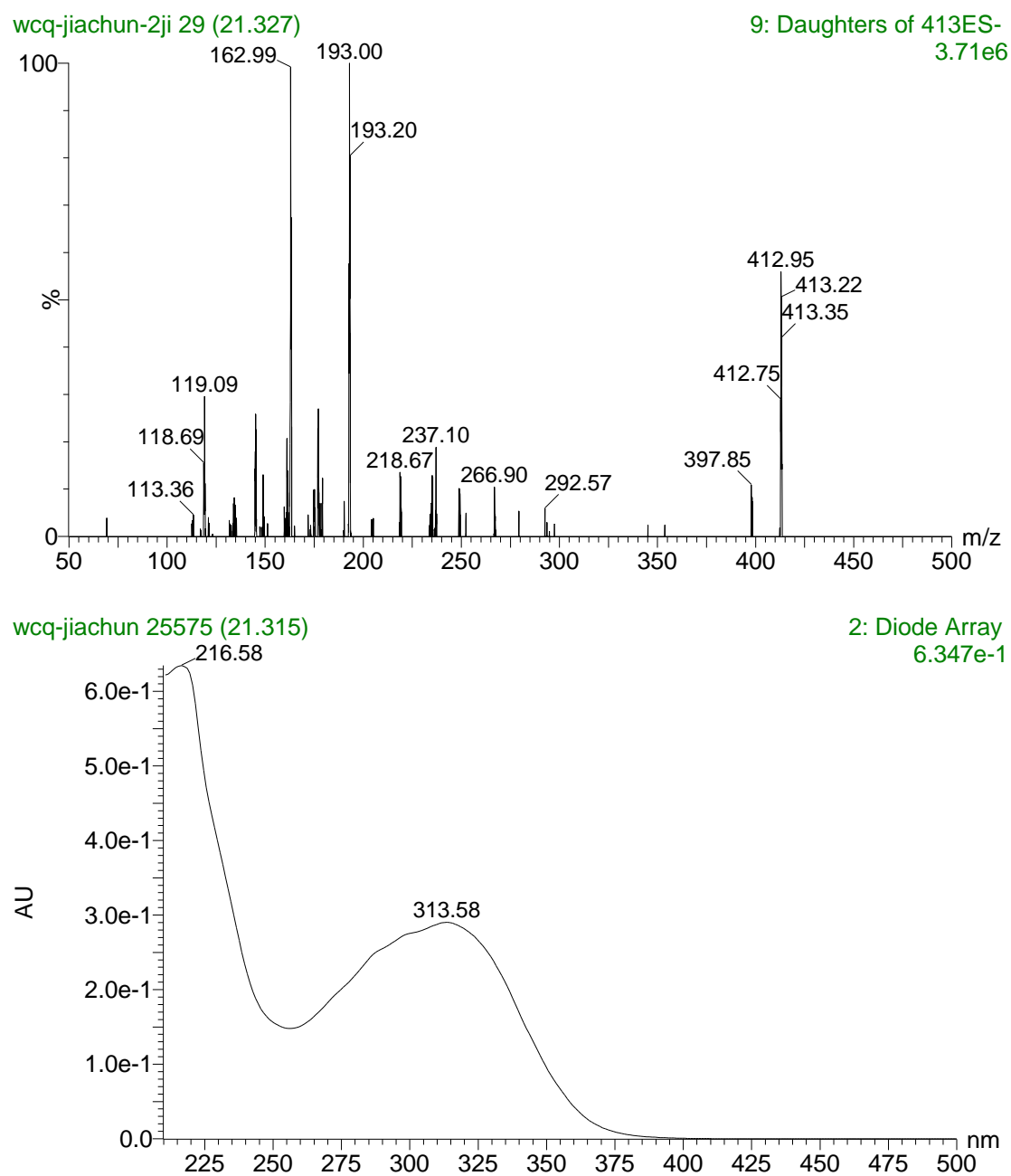

Figure S1. The MS/MS fragments and UV spectrum of 1-*O*-feruloyl-3-*O*-*p*-coumaroylglycerol.

Supplement: Supplementary file 1 [file foods-12-01144-s001.zip › Figure S1.pdf]
